# Single-molecule dynamics suggest that ribosomes assemble at sites of translation in *Bacillus subtilis*

**DOI:** 10.3389/fmicb.2022.999176

**Published:** 2022-11-03

**Authors:** Joshua Stoll, Victor Zegarra, Gert Bange, Peter L. Graumann

**Affiliations:** Centre for Synthetic Microbiology (SYNMIKRO) and Fachbereich Chemie, Philipps-Universität Marburg, Marburg, Germany

**Keywords:** bacterial cell biology, *Bacillus subtilis*, translation, GTPase, ribosome assembly, single molecule tracking, nucleoid occlusion

## Abstract

Eukaryotic cells transcribe ribosomal RNA and largely assemble ribosomes in a structure called the nucleolus, where chromosomal regions containing rRNA operons are clustered. In bacteria, many rRNA operons cluster close to the origin regions that are positioned on the outer borders of nucleoids, close to polar areas, where translating 70S ribosomes are located. Because outer regions of the nucleoids contain the highest accumulation of RNA polymerase, it has been hypothesized that bacteria contain “nucleolus-like” structures. However, ribosome subunits freely diffuse through the entire cells, and could thus be assembled and matured throughout the non-compartmentalized cell. By tracking single molecules of two GTPases that play an essential role in ribosomal folding and processing in *Bacillus subtilis*, we show that this process takes place at sites of translation, i.e., predominantly at the cell poles. Induction of the stringent response led to a change in the population of GTPases assumed to be active in maturation, but did not abolish nucleoid occlusion of ribosomes or of GTPases. Our findings strongly support the idea of the conceptualization of nucleolus-like structures in bacteria, i.e., rRNA synthesis, ribosomal protein synthesis and subunit assembly occurring in close proximity at the cell poles, facilitating the efficiency of ribosome maturation even under conditions of transient nutrient deprivation.

## Introduction

Assembly of ribosome subunits requires the orchestration of RNA processing, of ribosomal RNA folding and binding of a large number of proteins to the two subunits, as well as rRNA modifications such as base methylation, ribose methylation or pseudouridylation (Kressler et al., 2010; Trinquier et al., 2020). This complex process leads to multiple ribosomal intermediates in which the subunits are bound to assembly-related enzymes, and occurs within a sub-compartment in eukaryotic cells, the nucleolus. It has been unclear if non-compartmentalized bacteria might also employ subcellular organization of subunits assembly.

Proper ribosome assembly is essential for the viability of all cells, and is monitored by several factors, including a conserved family of GTPases, which play an essential role in ribosome maturation in prokaryotes and eukaryotes (Karbstein, 2007; Britton, 2009). Interestingly, bacteria appear to involve more GTPases, most of which were proposed to confer checkpoint-like functions, in ribosome assembly than eukaryotes. In *B. subtilis,* Obg, which is a member of the OBG-HflX-like superfamily, and Era, of the TrmE-Era-EngA-YihA-Septin-like superfamily, are two GTPases essential for viability (Trach and Hoch, 1989; Morimoto et al., 2002). Both superfamilies are part of the translation factor (TRAFAC) GTPase class, which belong to the larger superclass of P-loop GTPases. These contain a Walker A motif or P-loop (phosphate-binding loop) that predominantly binds to nucleotides (Verstraeten et al., 2011). Besides their affinity for GTP, these proteins are also able to bind to guanosine-based alarmones (p)ppGpp, allowing the cell to sense nutrition deprivation and act accordingly by downregulating GTP-consuming processes (Hamel and Cashel, 1974; Corrigan et al., 2016; Diez et al., 2020). Obg binds to the pre-50S ribosomal subunit, which has been shown by coelution of Obg together with the large subunit (Scott et al., 2000). Obg-GTP preferably binds to the 50S pre-state, while Obg-GDP gets released from the fully assembled subunit, suggesting that the regulation of Obg in large subunit maturation is GTP-dependent (Zhang and Haldenwang, 2004).

The *E. coli* Ras-like GTPase Era is involved in the maturation process of the pre-30S ribosomal subunit. It consists of a RNA-binding K homology domain and a GTP-binding domain (Tu et al., 2009). While bound to GTP, its conformation is suitable to bind to the pre-30S ribosomal subunit and to a conserved sequence (GAUCACCUCC) at the 3′-end of the pre-16S rRNA (Tu et al., 2011). In this complex the pre-16S rRNA is matured by RNase E, G and an unknown nuclease, which could be YqgF (Kurata et al., 2015; Kurata et al., 2018). After the hydrolysis step GDP is released from this complex (Tu et al., 2009). Structural data suggest that in *E. coli* the unbinding of Era is necessary for ribosomal protein S1 to consecutively bind to this region as both proteins would overlap (Sharma et al., 2005).

Ribosomal protein L1, part of the large subunit and conserved within all three domains of life (Roberts et al., 2008), was found to be nonessential in *B. subtilis* (Akanuma et al., 2012). In organisms with a very small genome, its encoding gene, *rplA*, can be lacking (Galperin et al., 2021). The loss of *B. subtilis rplA* leads to a slower growth rate, 70S formation is impaired, and concomitantly, an accumulation of 30S and 50S subunits occurs. Furthermore, sporulation frequency is reduced in an *rplA* deletion mutant (Akanuma et al., 2012). The product of the *B. subtilis ypfD* gene consists of four S1 domains, and is thus similar to *E. coli* S1 (EcS1) protein. In contrast to *Ec*S1, the YpfD protein does not co-purify with ribosomes (Isono and Isono, 1976; Hahn and Stiegler, 1986; Nanamiya et al., 2004), and it has thus been unclear if the protein is associated with ribosomes and translation. By showing that the *ypfD* gene product colocalizes with ribosomes and changes its single-molecule dynamics in response to transcription or translation arrest in a manner analogous to L1, we suggest that it is a functional ortholog of S1, but more loosely associated with the ribosome than *Ec*S1. We therefore propose to name YpfD “*Bs*S1,” which is done henceforth in this article. Noted as non-essential, it was shown that a strain lacking *ypfD* has a decrease in sporulation at high temperatures (Ohashi et al., 2003).

In eukaryotes the assembly of the ribosomes is spatially highly organized around the nucleus. Inside of the nucleoplasm Polymerase II synthesizes mRNA and it is exported to the cytoplasm where, in a close proximity, ribosomes translate these mRNAs into, e.g., ribosomal proteins. A fraction of the proteins is transported into the nucleolus by nuclear transport factors where they assembly together with newly synthesized immature rRNAs. Assisted by shuttling factors these intermediates of the small and the large subunit leave the nucleus. In the last steps of the maturation the shuttling factors dissociate from the complex and the last ribosomal proteins bind (Greber, 2016).

Prokaryotes do not contain a nucleus, which spatially regulates processes regarding DNA and its transcription into RNA. However, many bacteria contain so-called “nucleoids” where DNA is compacted in the more central part of the cell. In this region, RNA polymerases are actively transcribing, and there is less or no DNA close to the cell membrane than towards the center. This leads to an overall separation of translation and transcription in, e.g., *E. coli* and *B. subtilis* cells (Lewis et al., 2000; Mascarenhas et al., 2001). Due to its high negative charge, RNA is likely excluded from the nucleoid region in a fast manner [timescale of approximately 100 ms (Mohapatra and Weisshaar, 2018)]. On the other hand, transcription and translation can be tightly coupled for many genes (Merino and Yanofsky, 2005; O'Reilly et al., 2020); such coupling may predominantly occur at the boundary between nucleoids and surrounding translation zones. Fluorescence microscopic studies on ribosomal proteins showed a clear localization pattern for the ribosomal proteins L1 and S2 in the subpolar region of *B. subtilis* as well as an exclusion from stained DNA material (Lewis et al., 2000; Mascarenhas et al., 2001). This so-called “nucleoid occlusion” (NO) depends on active transcription, implying that RNA synthesis and thus the presence of mRNA/rRNA sets up a pseudo-compartmentalized location containing translating ribosomes and their substrate.

Interestingly, bacteria contain a large number of rRNA operons close to the origin regions on the chromosome. In *E. coli*, even distant rRNA operons cluster with most other operons in space (Gaal et al., 2016), accompanied by a major accumulation of RNA polymerases (RNAP) at these subpolar regions, during rapid growth conditions. These regions are located at the outer edges of the nucleoid(s; Bakshi et al., 2012; Stracy et al., 2015; Jin et al., 2017) and have been termed “nucleolus-like” structures. RNAP clusters have been shown to have liquid–liquid phase separation [LLPS (Su et al., 2021)] properties and involve antitermination factors, such as NusB (Ladouceur et al., 2020). Thus, LLPS appears to be a mechanism of subcellular organization in bacteria. It has been unclear if rRNA being synthesized close to the cell poles would also mature into ribosome subunits at polar regions, where ribosomal proteins are being synthesized, or if maturation of rRNA into subunits occurs throughout the cells. These are two different concepts: free ribosomal subunits freely diffuse through cells and do not show NO like translating ribosomes (Sanamrad et al., 2014), so maturing subunits could likewise show free diffusion. In this scenario, we would expect GTPases that are bound to maturing subunits (Scott et al., 2000; Tu et al., 2011), which should have a much lower diffusion constant than non-bound GTPases, to also show diffusion throughout the cell. In the alternative concept, GTPases would show confined diffusion at some subcellular space in the bacterial cell, if maturation of ribosome subunits occurs, e.g., in close proximity to sites of translation of ribosomal proteins, or close to sites of rRNA transcription.

In our work, we have followed the localization and dynamics of Era and Obg in *B. subtilis* cells using single-molecule tracking. We characterize the change in their dynamics in response to mRNA depletion, arrest in translation and during the stringent response. While ribosomal subunits freely diffuse throughout the cytosol, slow-moving GTPases, assumed to be in complex with maturing ribosomal subunits, show NO, and respond to translation stress similar to ribosomes. We also show that a putative *B. subtilis* S1 protein shows dynamics closely resembling those of L1, indicating that S1 is indeed associated to the ribosome during translation.

## Results

### Era and Obg can be expressed as functional C-terminal mVenus fusions

We integrated C-terminal fluorescent protein (mVenus) fusions at the original gene loci (i.e., under control of the original promoter) for Era, Obg, L1 and for *Bs*S1. All strains grew indistinguishable from cells not carrying a fluorescent protein fusion (Supplementary Figure S1A), suggesting that Era-mVenus, Obg-mVenus and L1-mVenus can functionally replace the corresponding wild type protein. The deletion of *ypfD* (encoding for *Bs*S1) does not result in any discernable phenotype (Sorokin et al., 1995), so we cannot judge if the fusion protein is functional. As will become apparent later, the protein shows dynamics very similar to those of L1, indicating that the fusion protein retains its activity. Western blotting showed that L1-mVenus, Era-mVenus, Obg-mVenus and *Bs*S1-mVenus fusions were expressed as full-length fusions (Supplementary Figure S1B). Of note, detection of fluorescent-protein tags is more efficient in our hands when samples with added SDS loading buffer are not boiled. Therefore, all fusions run slightly faster than expected from their size.

The depletion of Era and Obg in *E. coli* or in *B. subtilis* leads to a block in chromosome replication and/or in chromosome segregation (Kok et al., 1994; Kobayashi et al., 2001), resulting in the formation of highly elongated cells. Cell length distribution showed a 10% increase in average cell lengths for Obg, less for L1 and BsS1-mVenus fusion strains, and none for the Era fusion strain (Supplementary Figure S2, Table 1), compared to 2.77 μm for cells lacking any protein fusion. Bearing in mind that the addition of a 28 kDa fusion protein usually affects full functionality of any protein, absence of a detectable growth defect or considerable effect on cell division shows that fusions largely take over wild type functions.

**Table 1:** Statistical data from single-molecule experiments of *B. subtilis* mVenus-fusion strains using SQD analysis.

	**Era-mV**	**Obg-mV**	***Bs*S1-mV**	**L1-mV**
**exponential growth**
# tracks	2,319	5,357	3,957	5,688
Av. cell length [μm]	2.83	3.07	3.02	3.04
R^2^(1 frame)	0.99891	0.99889	0.99772	0.99885
pop1 [%]	37.2 ± 1.0	42.6 ± 0.5	59.6 ± 0.6	58.6 ± 0.5
pop2 [%]	62.8 ± 1.0	57.4 ± 0.5	40.4 ± 0.6	41.4 ± 0.5
D1 [μm^2^sˉ^1^]	0.081 ± 0.003	0.084 ± 0.001	0.052 ± 0.0007	0.056 ± 0.0006
D2 [μm^2^sˉ^1^]	0.57 ± 0.01	0.77 ± 0.008	0.59 ± 0.02	0.54 ± 0.009
**+ rifampicin**
# tracks	3,845	2,348	5,479	4,186
Av. cell length [μm]	2.66	2.94	3.06	3.00
R^2^(1 frame)	0.99952	0.99849	0.99927	0.99939
pop1 [%]	27.0 ± 0.5	38.7 ± 0.9	21.3 ± 0.3	27.6 ± 0.6
pop2 [%]	73.0 ± 0.5	61.3 ± 0.9	78.7 ± 0.3	72.4 ± 0.6
D1 [μm^2^sˉ^1^]	0.16 ± 0.003	0.11 ± 0.003	0.11 ± 0.002	0.14 ± 0.003
D2 [μm^2^sˉ^1^]	0.83 ± 0.006	0.73 ± 0.01	0.91 ± 0.004	0.68 ± 0.005
**+ chloramphenicol**
# tracks	1861	4,239	5,665	7,189
Av. cell length [μm]	2.85	3.07	3.25	3.02
R^2^(1 frame)	0.99847	0.99822	0.99811	0.99869
pop1 [%]	35.1 ± 0.7	44.6 ± 0.8	58.4 ± 0.5	53.0 ± 0.4
pop2 [%]	64.9 ± 0.7	55.4 ± 0.8	41.6 ± 0.5	47.0 ± 0.4
D1 [μm^2^sˉ^1^]	0.081 ± 0.002	0.067 ± 0.002	0.042 ± 0.0005	0.052 ± 0.0006
D2 [μm^2^sˉ^1^]	0.67 ± 0.01	0.72 ± 0.02	0.56 ± 0.01	0.56 ± 0.009
**+ serine hydroxamate**
# tracks	7,722	15,030	3,395	7,236
Av. cell length [μm]	2.95	2.91	3.11	3.10
R^2^(1 frame)	0.99914	0.99925	0.99840	0.99907
pop1 [%]	35.0 ± 0.3	29.9 ± 0.2	55.1 ± 0.8	50.9 ± 0.6
pop2 [%]	65.0 ± 0.3	70.1 ± 0.2	44.9 ± 0.8	49.1 ± 0.6
D1 [μm^2^sˉ^1^]	0.085 ± 0.001	0.079 ± 0.0007	0.062 ± 0.001	0.069 ± 0.0009
D2 [μm^2^sˉ^1^]	0.8 ± 0.005	0.92 ± 0.003	0.68 ± 0.02	0.51 ± 0.008

**Figure 1:**
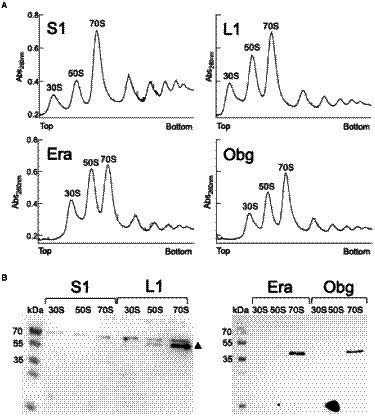
**(A)** Ribosome profiles of exponentially growing *B. subtilis* cells expressing mVenus fusions to the proteins indicated within the profiles. Peaks containing individual ribosomal proteins, translating 70S ribosomes or polysomes are indicated above the peaks. **(B)** Western blots using anti-GFP antiserum show the presence of L1-mVenus (52 kDa) within 50S and 70S peaks (indicated by a black triangle); the band at 70 kDa is a non-specific band possibly masking the presence of *Bs*S1-mVenus (70 kDa). Era-mVenus (61 kDa) or Obg-mVenus (74 kDa) are not visibly associated with mature subunits or translating ribosomes.

We tested if L1-mVenus or *Bs*S1-mVenus are incorporated into ribosomal subunits. Figure 1 shows fractionation experiments revealing that L1-mVenus is found in 50S subunits and in 70S ribosome fractions, but not in the small subunits. Because we obtained a non-specific band at 70 kDa, we could not determine if *Bs*S1 is incorporated into small subunits. For Era-mVenus and Obg-mVenus, we did not expect stable incorporation into ribosomal subunits, as they are associated with assembly intermediates, and most likely in a highly transient manner. Indeed, we did not observe any specific band for the two GTPases, in contrast to L1-mVenus (Figure 1B). For all strains expressing the fusion proteins, ribosome profiles showed an expected pattern of free subunits versus 70S and polysome populations (Figure 1A), similar to that of cells lacking a protein fusion (Supplementary Figure S3), in agreement with the wild type-like growth (Supplementary Figure S1A). These experiments suggest that all fusions, expressed as sole source of the proteins, fulfill their corresponding essential (Era, Obg) or important roles; we carried on investigating *Bs*S1 keeping in mind the fusion might not be functional.

### GTPase-mVenus fusion do not show visible accumulations like ribosomal proteins using epifluorescence microscopy

**Figure 2:**
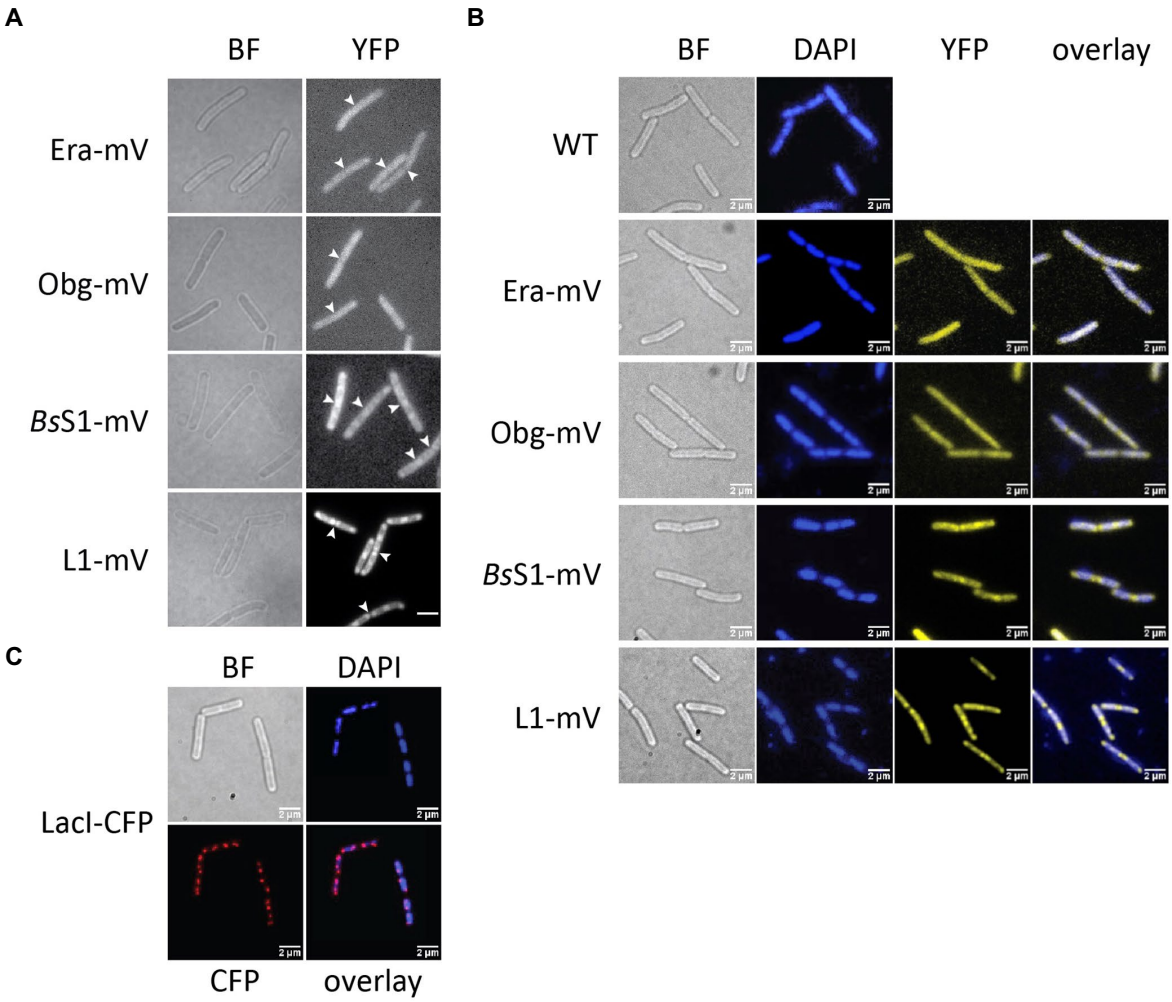
Epifluorescence experiment of exponentially growing *B. subtilis* cells expressing C-terminal mVenus protein fusions from the original gene loci. **(A)** Left panels show brightfield (BF) images of cells, right panels display the corresponding epifluorescence micrographs (“YFP,” same exposure times). Nucleoids are apparent in fluorescent micrographs from ribosomal protein fusions as areas from which ribosomes are largely excluded. Ends of cells are highlighted by white arrows. **(B)** Live cells in which nucleoids are stained with DAPI, “overlay” shows merge of YFP and DAPI channels. **(C)** Strain carrying a *lacO* cassette close to origin regions on the chromosome, to which LacI-CFP binds, expressed from a constitutive promoter. Scale bars 2 μm.

Epifluorescence experiments showed that L1-mVenus localizes to sites surrounding the nucleoids, i.e., mostly at the cell poles or at future division sites between two nucleoids (Figures 2A,B), similar to what has been described for ribosomes in *E. coli* and in *B. subtilis* cells (Lewis et al., 2000; Mascarenhas et al., 2001; Bakshi et al., 2012). These data suggest that L1-mVenus is largely incorporated into ribosomes, while non-incorporated protein would localize throughout the cells. For *Bs*S1-mVenus, we found much weaker fluorescence compared to L1-mVenus, but a highly similar localization pattern (Figure 2A), both proteins localized around the centrally located nucleoids (Figure 2B). NO of *Bs*S1 suggests that it may be closely associated with ribosomes *in vivo*.

For Era-mVenus and Obg-mVenus, we found only very weak fluorescence (note that intensity has been highly increased relative to *Bs*S1 and L1 panels, in order to see some signal), which was distributed throughout the cells (Figure 2A). These observations suggest that both GTPases are expressed at very low levels compared to L1, in agreement with Western blot analyses (Supplementary Figure S1B). Nucleoid morphology of the strains expressing ribosomal proteins was indistinguishable from that of wild type strains (Figure 2B), suggesting that fusions do not interfere with normal chromosome organization. As described before, most cells contained two separated origin regions, as visualized using a LacI-*lacO* system, which were located at the outer edges of the nucleoids (Figure 2C). Origin regions are surrounded by 7 out of 10 rRNA and ribosomal protein operons, and only 3 operons are located further within the nucleoid area (Davies and Lewis, 2003). Thus, most rRNA synthesis occurs close to polar sites of translation, in agreement with an accumulation of RNA polymerase at these sites (Lewis et al., 2000), likewise to what was described for *E. coli* (Gaal et al., 2016). However, epifluorescence microscopy employs an even illumination across a wide field of view, requiring exposure times of 500 ms (for ribosomal proteins) or 2000 ms (GTPases) to obtain sufficient fluorescence signal. These time regimes blur out diffusive motion of molecules. As will become apparent in the following section, the localization of GTPases is indeed blurred out in epifluorescence acquisitions, masking a preferred slow diffusion at subcellular sites (see below).

### Single-molecule tracking reveals nucleoid occlusion of active GTPases is dependent on active transcription

Single-molecule tracking (SMT) was done using slim-field illumination. Briefly, during the first frames (500 for GTPases and *Bs*S1-mVenus, 1,000 for L1-mVenus) molecules bleach, until few to single molecules remain (characteristically where the slope of the bleaching curve has reached a slope of 10% or less) and their movement is monitored for an additional 2000 frames (yielding average track lengths of about 8 steps). Observed trajectories were automatically tracked using u-track (Jaqaman et al., 2008). All SMT data were analysed using SMTracker 2.0 (Oviedo-Bocanegra et al., 2021), only tracks of 5 steps or more were considered to avoid a bias by very short events of (non)-motion.

**Figure 3:**
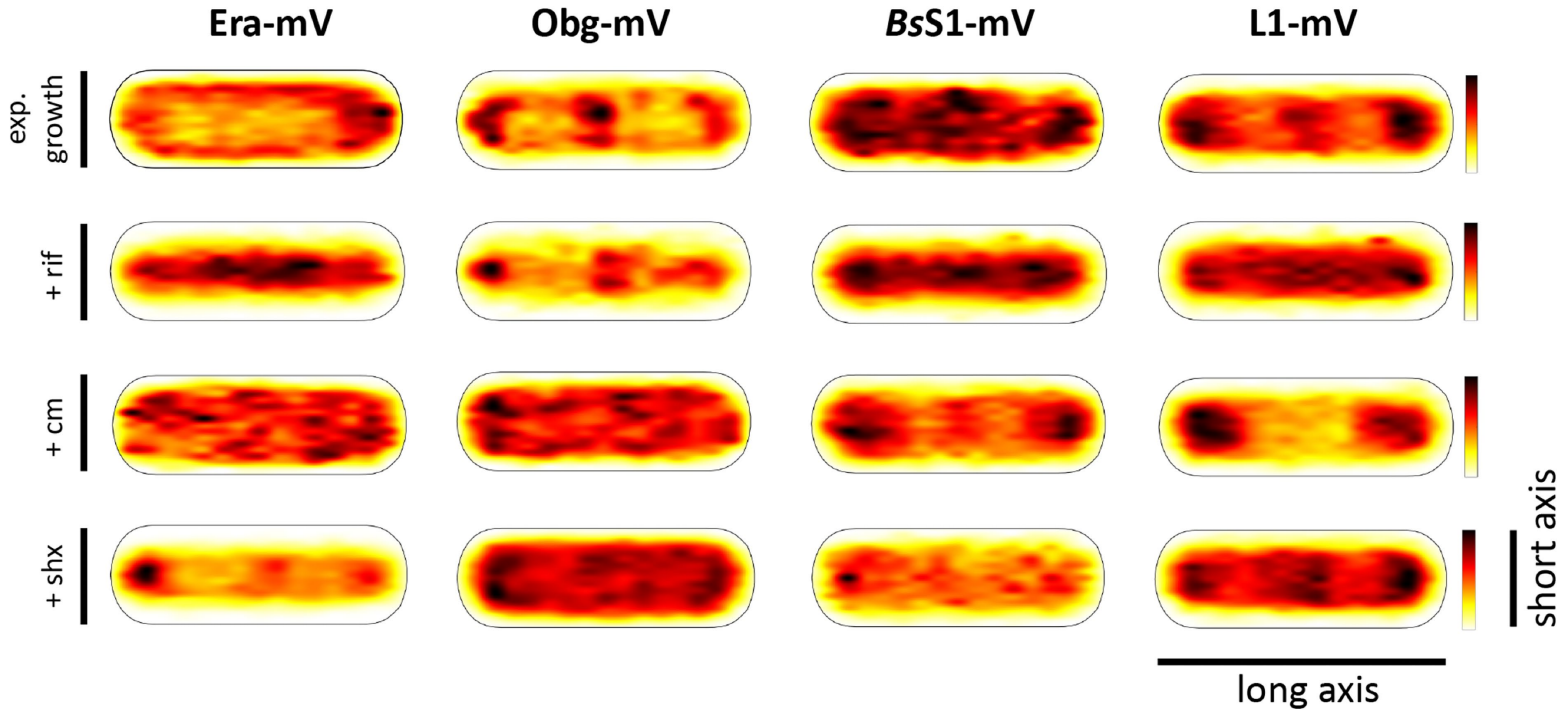
Heat maps of all trajectories projected into a single average-sized cell (1 × 3 μm). Scales are relative abundance of proteins, with higher (darker colours) or lower (yellow) probability of where trajectories of fluorescent fusion proteins are observed.

Strikingly, when all tracks of Era and Obg were projected into an average-sized *B. subtilis* cell of 1 × 3 μm (note that cells are actually thinner with 0.75 μm), a clear preference for molecule localization at subcellular sites surrounding the nucleoids, most predominantly at the cell poles, is apparent (Figure 3). For L1, clear localization in an NO manner is visible, and also for *Bs*S1, although less pronounced than for L1. Note that accumulation of Obg, L1, and *Bs*S1 in the cell middle is due to large cells containing two segregated nucleoids, where there is new space for translating ribosomes. Why this is not observed for Era is somewhat puzzling, but interesting to note. These data suggest that (a) *Bs*S1 is a part of translating ribosomes, but likely more loosely associated (i.e., more freely diffusive) than previously described ribosomal proteins, and (b) Era and Obg are mostly engaged in ribosome-associated assembly processes at sites where ribosomes are mostly present (Lewis et al., 2000).

**Figure 4:**
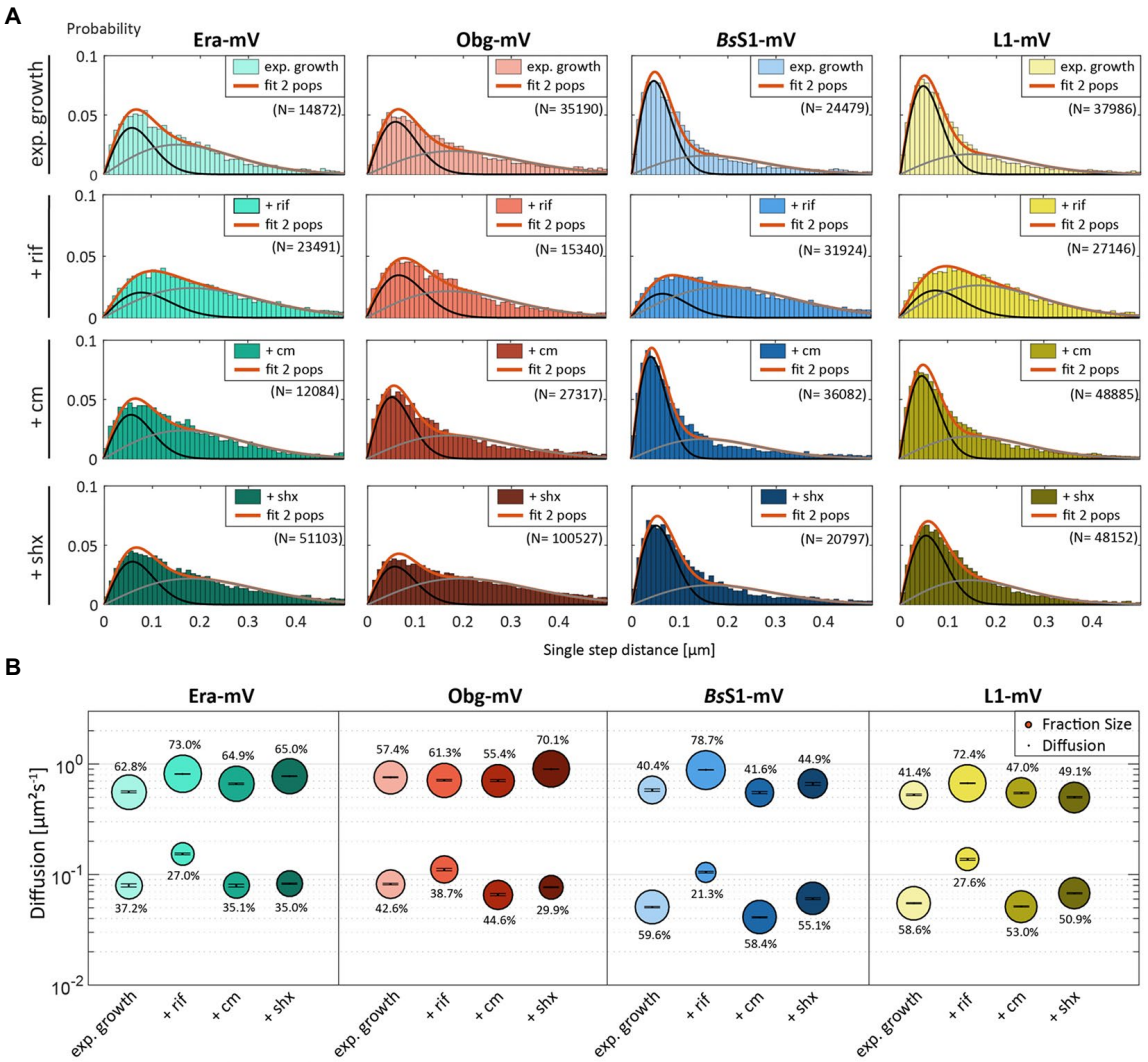
Single-molecule tracking of C-terminal mVenus fusions of the GTPases Era and Obg and the ribosomal proteins L1 and *Bs*S1 during exponential growth. For single-molecule experiments during physiological perturbations, cells were treated with rifampicin or chloramphenicol for 30 min, or with or DL-serine hydroxamate for 10 min prior to tracking. **(A)** Using jump distance analyses the resulting data were analysed and the probabilities of single step distances plotted up to a maximum value of 0.5 μm. The two-population fit (orange curve) together with its static (dark grey) and mobile (light grey) population suited the data well. **(B)** Bubble plots represents the D of the population on the y-axis by height and population size by the bubble diameter. Corresponding values can be found in Table 1.

In order to test if ribosome assembly truly occurs at sites of active translation, we treated cells with a subinhibitory concentration of rifampicin (40 μg/ml) that did not lead to visible cell death after 30 min [full inhibition at 100 μg/ml (Price and Frabotta, 1972) leads to extremely bright fluorescence in >40% of treated cells]. Figure 4A shows that step lengths of molecules (for more details see next section) increased markedly for L1-mVenus. Figure 4B shows that D of mobile fractions of L1 and of *Bs*S1 almost doubled (likely depletion of polysomes), and the size of the mobile free subunit populations increased at the expense of the slow mobile populations, as expected from a strong reduction in mRNA levels. NO became much less pronounced, L1 molecules now occupied all central spaces of the (tube-like) cells (Figure 3, second row). Likewise, slow mobile/static fractions of GTPases showed higher diffusion constants and decreased sizes (Figure 4B). Diffusion constants for mobile fractions all increased, which we have seen for other cytosolic proteins (Rotter et al., 2021), indicating that loss of NO generally speeds up molecule diffusion in bacterial cells. GTPases also completely (Era) or largely (Obg) lost diffusion in a nucleoid occlusion-dependent manner (Figure 3).

As an additional control, we treated cells with chloramphenicol, which inhibits the peptidyl transferase center and thus stalls ribosomes on mRNA. This treatment indeed “froze” L1 and *Bs*S1 populations in their steady state diffusion conditions (note that NO for *Bs*S1 appeared visually more pronounced, Figure 3), but lead to less pronounced NO for Era and for Obg, in agreement with fewer, newly synthesized ribosomal proteins, cutting down subunit assembly intermediates as substrates for GTPases.

These experiments show that NO for GTPases depends on steady state mRNA synthesis and is influenced by ribosome activity.

### Single-molecule dynamics of GTPases resemble those of ribosomes

As opposed to an in-depth characterization of single-molecule dynamics of GTPases, for which different acquisition times would be advisable, we chose to use a single integration time in order to compare dynamics of Era and of Obg with those of ribosomes, represented by L1 and *Bs*S1. Figure 4A shows jump distance analyses of the four proteins, which is based on squared distance analysis (SQD). The probability of jumps was fitted using two Rayleigh distributions, which could explain the observed distribution very well, yielding R^2^ values above 0.99 (Table 1). The analyses for L1 were challenging due to a considerable number of free mVenus molecules (Supplementary Figure S1B) which may convolute the data. However, accurately tracking mVenus (having a D of about 5 μm^2^/s) requires 5 ms integration time (Schibany et al., 2018) and at the 20 ms used in this study, only very slow-moving mVenus molecules would be captured and not blurred out. Tracking data of *Bs*S1-mVenus are very similar to those of L1-mVenus (Figure 4A), therefore we believe that L1-mVenus data are only convoluted to a negligible degree. Considering diffusion constants shown in Figure 4B and in Table 1, we propose that the populations of L1-mVenus and of *Bs*S1-mVenus with the lowest diffusion constants (of 0.052 or 0.056 μm^2^/s) represent L1 or *Bs*S1 in ribosomes being engaged in translation, i.e., 70S ribosomes and polysomes. This would be in agreement with earlier analyses of ribosomes in *E. coli,* where Ds of 0.04 to 0.055 μm^2^/s were determined (Bakshi et al., 2012; Sanamrad et al., 2014). Note that errors given in Table 1 are fitting errors, which are determined using “the cross validation” tool in SMTracker 2.0. This tool compares errors derived from a training set (split into random 10% sets for each of which a fit is performed) and a test set of the other 30% of data, in order to avoid overly small errors derived from very large data sets.

The mobile fraction had a D of 0.54 μm^2^/s for L1 and 0.59 for *Bs*S1 (Table 1), similar to that of free subunits determined in *E. coli* (0.4 μm^2^/s; Sanamrad et al., 2014). A possible, freely diffusing population of L1-mVenus or of *Bs*S1-mVenus is likely not captured using 20 ms integration time, as the D-values of small cytosolic proteins in *B. subtilis* are usually between 1 and 2 μm^2^/s (Rosch et al., 2018; Schibany et al., 2018; Romero et al., 2019). Figure 4B shows that about 60% of L1 or *Bs*S1 were found in the putative 70S/polysome fraction and 40% in the 30S/50S subunit fraction, somewhat different from an 85%/15% ratio between engaged and free subunits determined by sucrose gradient centrifugation of purified ribosomes (Forchhammer and Lindahl, 1971; Mawn et al., 2002), but comparable to an SMT study on *E. coli* ribosomes (Bakshi et al., 2012). Due to the complexity of the process of translation (initiation, elongation, termination, recycling), the real number of populations is likely higher, interchangeable and very dynamic. However, for the purpose of distinguishing between GTPases engaged (bound to subunits or intermediate states) or not engaged (freely diffusive) in maturation, assuming two populations seems appropriate.

Interestingly, diffusion constants for the static fractions of Era and Obg (0.081 or 0.084 μm^2^/s) were similar to those of L1 and *Bs*S1, yet 1.5-fold higher (Figure 4B). Because bacterial GTPases involved in ribosome biogenesis are not known to be active components of translating ribosomes (except for a possible involvement in degradation, see discussion), we favour the idea that 37% or 43% of Era or Obg molecules, respectively, are engaged with slow-moving, maturing ribosome subunits. About 63% of Era molecules have a D very close to that of the mobile fractions of L1 and *Bs*S1, while the D for the mobile Obg fraction of 57% is higher at 0.77 μm^2^/s. We did not detect a mobile fraction of GTPases having a D higher than “1,” which would be expected for freely diffusive, average sized cytosolic proteins, suggesting that these GTPases are largely present in multiprotein complexes. Alternatively, they could be rapidly changing between binding to complexes, thereby moving in a constrained fashion, in analogy to DNA binding proteins moving through the nucleoid in a similar manner.

### Stringent response leads to a mild shift in ribosome populations and only affects Obg dynamics

We wished to analyse to what extent ribosome and GTPase dynamics are affected by amino acid starvation. We treated cells with DL-serine hydroxamate (SHX), which blocks serine tRNA synthetase activity, depleting cells of charged serine tRNAs. This induces the stringent response, where a RelA-dependent increase in (p)ppGpp levels leads to strong changes in ribosomal RNA synthesis, besides many other physiological adaptations (Steinchen and Bange, 2016). We chose a concentration of SHX (30 mM) that leads to a slowed-down, but still measurable cell growth, in order to avoid artefacts through growth inhibition (Hernandez-Tamayo et al., 2021). Interestingly, we only observed a mild shift of slow-mobile molecules towards mobile L1 and *Bs*S1 molecules (Figure 4B), and a mild increase in their diffusion constants. This indicates that while the synthesis of new ribosomes is reduced, due to a shut-down of translation and of rRNA synthesis (Steinchen and Bange, 2016), steady state populations are mainly kept constant. Note that many GTPases involved in translation are inhibited by (p)ppGpp (Hamel and Cashel, 1974; Diez et al., 2020), such that translation rates will be slowed down.

For Era, we did not observe a strong change in its dynamics, but we observed a pronounced shift of static/slow mobile molecules towards mobile molecules for Obg. While NO remained visible for all four proteins, Era showed a visually less pronounced NO compared with exponential growth conditions (Figure 3). Thus, although GTPases are inhibited by (p)ppGpp binding (Corrigan et al., 2016; Pausch et al., 2018), they appear to continue to be associated with polar assembly of ribosome subunits under the sub-inhibitory conditions used in our study.

### Confined motion of assembly GTPases colocalizes with areas of translating ribosomes

**Figure 5:**
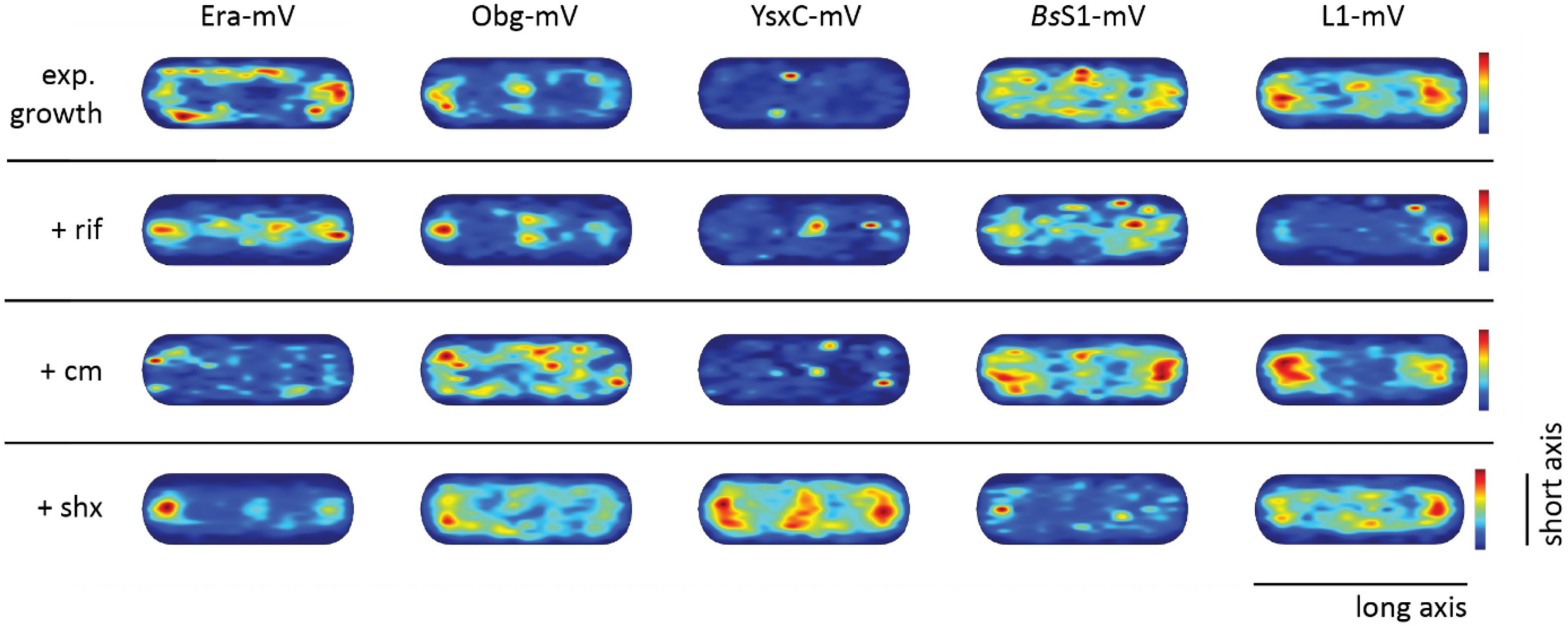
Confined motion maps derived from SMT experiments. Maps were created using the dwell radii representing three times the localization error from the corresponding dataset (Supplementary Table S1). Red to blue indicates higher to lower probability of confined motion. Due to a difference in the number of trajectories and more or less narrow spots of high probabilities there is a difference in the maximum value of the scale (Supplementary Table S1).

To visualize the different states of mobility of GTPases within cells, we generated heat maps for confined motion of molecules, representing subcellular locations where GTPases show dwell events, e.g., *via* binding to immature ribosomal subunits. We defined tracks that did not leave a specific diameter, derived from three times the localization error, for six steps or longer. Figure 5 indicates that Era and Obg showed similar high density of confined motion towards the cell poles and in the cell centre, as well as underneath the cell membrane. Era and Obg showed very similar NO of confined motion like *Bs*S1 and L1. For comparison, we also tracked GTPase YsxC-mVenus, for which a function has been proposed in 50S maturation, possibly in one of several parallel pathways (Ni et al., 2016). YsxC-mVenus showed a visibly weaker accumulation surrounding the nucleoids than Era or Obg, but clearly a NO pattern (Figure 5). Supplementary Table S1 shows that intensity values of YsxC are higher than those of Era or Obg, i.e., light blue value of YsxC are similar to yellow/red values of Era. This means that there is a considerable difference between dark blue and light blue values, revealing that YsxC is depleted from nucleoid areas (Figure 5). Thus, NO localization is conserved between the three investigated GTPases, but to different degrees.

During inhibition of transcription, areas of confined motion underneath the cell membrane moved towards the cell center for Era, and likewise, NO localization for L1 and for *Bs*S1 was much less pronounced (Figure 5). For Obg, the pattern of confined motion did not change dramatically during RNAP inhibition.

Inhibition of translation retained or even exaggerated NO for ribosomal proteins (Figure 5). Different from this, the pattern of localization of confined tracks for GTPases was strongly changed, confined tracks were less often found at the cell poles/at sites surrounding the nucleoids. These findings indicate that a reduction in the supply of newly assembled ribosome subunits, in response to transcription and translation inhibition affects the location of biogenesis GTPase molecules, especially to those that are bound to large structures showing confined diffusion.

During the stringent response, NO for confined motion of ribosomes was retained (Figure 5), in agreement with the continued presence of slow-diffusing molecules representing actively translating ribosomes (Figure 4B). Interestingly, NO was also retained for both GTPases, which is consistent with the idea that translation and thus assembly of new ribosomes is possible during amino acid starvation. For YsxC-mVenus, induction of the stringent response led to a strong shift of confined motion towards polar and mid-cell regions, suggesting that the YsxC-dependent assembly pathway of 50S subunits plays a special role during this stress response, as opposed to exponential growth conditions. These analyses reinforce the idea that Era, Obg and YsxC are involved in the assembly of ribosome subunits at sites of translation, and do not operate in a freely diffusive manner.

### Nucleoid occlusion is maintained under slow-growth conditions

RNA polymerase has been shown to form foci close to the nucleoid borders in fast-growing cells, while it is relatively evenly distributed within the nucleoid during slow growth (Lewis et al., 2000; Jin et al., 2017). This has been interpreted as “transcription foci” arising in cells that grow towards maximum doubling time, where the synthesis of ribosomes is considered to be the highest energy-consuming process in the cell. We wished to analyse if exclusion of translating ribosomes from nucleoids also depends on rapid growth conditions and on high transcription rates of rRNA operons. For this purpose, cells were grown in medium containing sorbitol instead of glucose, where the doubling time is decreased to 185 min on average, from 93 min (Soufo et al., 2008) under normal growth conditions.

**Figure 6:**
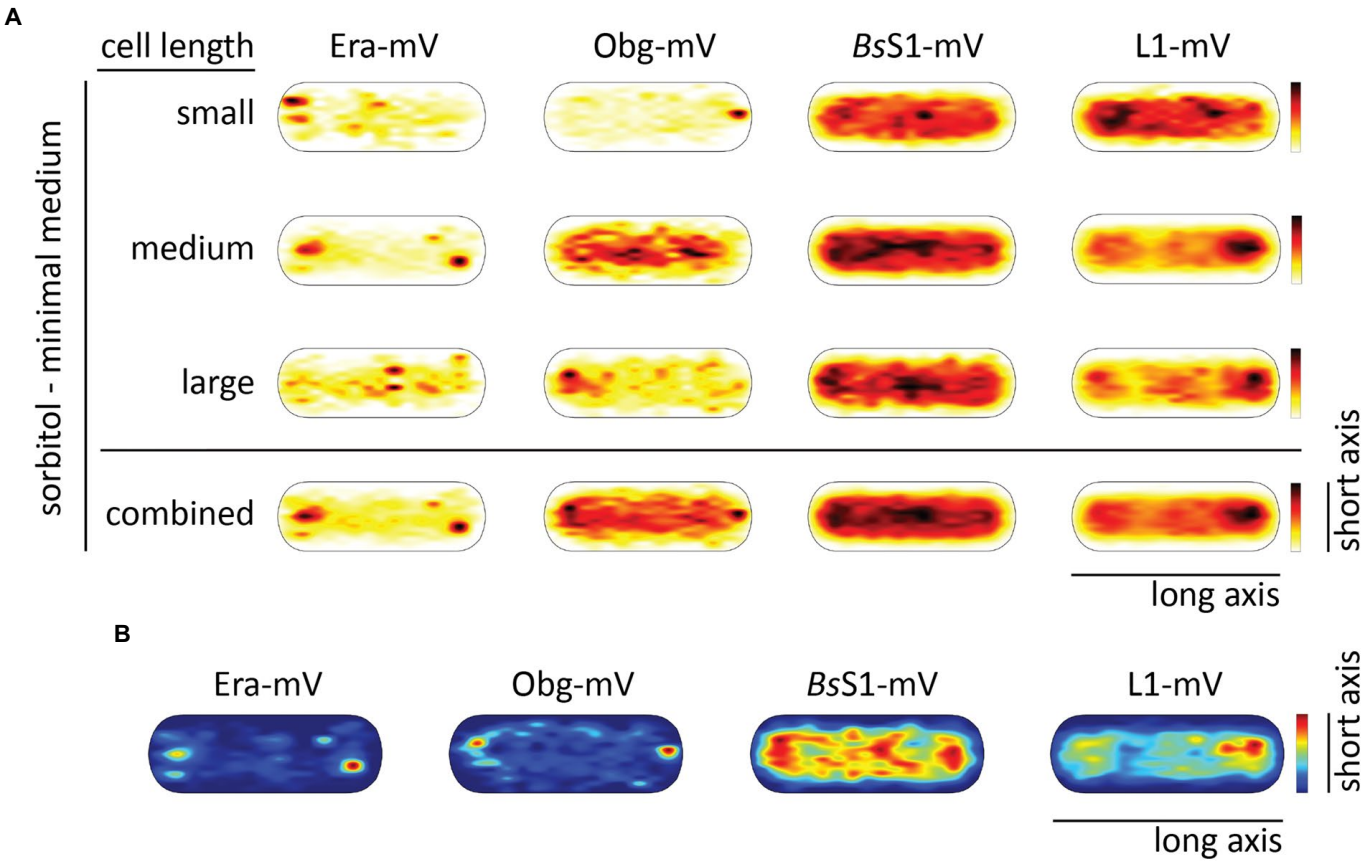
Heat maps showing nucleoid occlusion of GTPases and ribosomes under slow growth conditions. **(A)** Heat maps of the trajectories separated by a qualitative cell size give an example of how the molecule localization changes when larger cells possess two chromosomes. **(B)** Tracks that show confined motion within a certain dwell radius are most likely being bound to a larger complex and thus giving a hint to the point of action of the protein. The used dwell radii (which was set as three times the localization error) can be found in Supplementary Table S2. A variation in the maximum value of the scale results in a different coloration of the maps. For a better understanding in the case of a lighter overall coloration the maximum values are also given in Supplementary Table S2.

In Figure 6A, cells are divided into small cells, containing a single nucleoid, medium cells (usually containing a dumbbell-shaped nucleoid), and large cells, which usually contain two separated nucleoids. It is clearly visible that NO continues to dominate biogenesis GTPases as well as ribosome localization under slow growth conditions. Figure 6B shows that polar accumulation is largely based on confined motion of molecules at these subcellular spaces. Curiously, medium sized cells showed completely diffusive localization for Obg (note that cells have a shape of a cylinder), for which we do not have any explanation. However, the combined result also shows general NO for Obg, as for all other proteins investigated.

In accordance with the continued localization of the GTPases as well as the ribosomal proteins in a NO pattern, the distribution between fast- and slow-mobile fractions remained similar with regard to average diffusion constants and population sizes during exponential growth (Supplementary Figure S4; Supplementary Table S3). However, while the slow mobile fraction, likely accounting for 70S ribosomes or polysomes, retained its mobility, there was an increase in the diffusion constant of the fast-mobile fraction of the small subunit-associated proteins Era and *Bs*S1. Possibly, cellular crowding is lower during slow growth, leading to increased mobility of freely diffusing ribosomal subunits.

### An *era* allele with a mutation in the GTPase motif does not lead to stalling of ribosome biogenesis

In a previous work, it was shown that for *Ec*Era, the ability to hydrolyze GTP strongly relies on the conserved P-loop residue N18. When mutating this specific residue to alanine the proteins compromise their ability to hydrolyze GTP to GDP *in vitro*, which is dependent on the impaired binding ability to potassium cations (Rafay et al., 2012). We speculated that it would be likely that the loss in the activity of GTPases would disturb cell proliferation due to a lack of mature ribosomes. To investigate how *B. subtilis* can cope with an additional copy of Era having a reduced GTPase function *in vitro* we tested for a possible dominant negative effect of such an allele.

**Figure 7:**
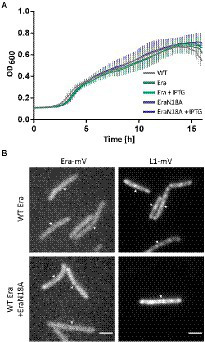
**(A)** Growth curves of *B. subtilis* BG214 and strains containing either a second wild type copy *Bs*Era or a an allele with a point mutation in the GTPase motif generating *Bs*EraN18A at 30°C upon induction with 1 mM IPTG (+ IPTG), or without induction as indicated in the inset. **(B)** Epifluorescence images of strains containing C-terminal mVenus-fusions of either Era or L1. Note that there is no untagged wildtype version of Era in the Era-mVenus and EraN18A strain. Scale bar 2 μm.

To gain more insight into the *in vivo* function of GTP-binding motif, we created a strain containing the inducible GTPase deficient EraN18A protein under the strong hyperspank promoter at the *amyE*-site. In order to investigate if a higher level of wild type Era *per se* has a negative effect on cells, we introduced a second copy of *Bs*Era under the same promoter. To visualize expression levels, we introduced a third construct, Era-mVenus into the same construct. Supplementary Figure S5 shows that induction with 1 mM IPTG resulted in massive expression of Era-mVenus, in contrast to barely detectable levels in cells expressing the fusion protein from the original gene locus. Interestingly, growth curves of all strains under maximum induction of the promoter showed similar growth to that of the *B. subtilis* BG214 wild type strain (Figure 7A). Also, the ability to form colonies was not affected (Supplementary Figure S6). This strongly suggests that (a) overproduction of Era does not affect ribosome assembly or any other essential aspect of the physiology of cells, and (b) the GTPase deficient protein does not stall ribosome maturation, because it was induced to vast excess over the wild type copy.

Furthermore, we tested if the localization of ribosomes might be perturbed, because GTPase deficient Era might not lead to a block in maturation, but affect NO localization of ribosomes. When the dynamics of the ribosome maturation process are only slowed down, rather than blocked, this could impact the localization patterns of GTPases and ribosomes. The diffuse signal of Era-mVenus as seen before (Figures 2A,B) did not change after induction of EraN18A, nor did L1-mVenus lose its NO localization pattern (Figure 7B). In case the generated mutation leads to the presumed loss of GTPase activity, our findings suggest that the binding of a GTPase deficient Era to the pre-mature 30S ribosomal subunit does not stall its further maturation.

## Discussion

The idea that bacteria contain a remarkable degree of subcellular organization in spite of generally lacking internal membrane systems has found its way into textbooks. It is widely known that bacterial chromosomes have a preferred three-dimensional arrangement within cells, with genes and chromosome sites occupying preferred sites within cells for most of the cell cycle, which undergo a well-organized choreography during DNA replication and segregation (Graumann, 2014; Badrinarayanan et al., 2015; Pioro and Jakimowicz, 2020). The Z-ring composed of the tubulin ortholog FtsZ can find the cell middle to assemble into a ring structure for further recruitment of cell division proteins by several different spatial guiding systems, and the actin-like MreB forms filamentous structures perpendicular to the long axis of cells to arrange cell wall synthesis, while many protein complexes specifically assemble at the cell poles (Thanbichler, 2010). Moreover, while transcription takes place on the nucleoids, 70S ribosomes are accumulated at polar regions devoid of DNA, setting up a pseudo-nucleus structure in many bacterial species (Lewis et al., 2000; Bakshi et al., 2012).

In eukaryotic cells, rRNA genes are clustered within the nucleolus, which also hosts the assembly of ribosome subunits, as ribosomal proteins are transported into the nucleus to be assembled on rRNA within the nucleolus. Because most rRNA operons are located close to origin regions on the chromosome in many bacterial species, it has been proposed that nucleolus-like structures might exist in bacteria (Jin et al., 2017). For the most part of the cell cycle, these regions are localized to the outer edges of the nucleoids (Davies and Lewis, 2003), and contain a high concentration of RNA polymerase (Lewis et al., 2000; Stracy et al., 2015), as well as transcription factor NusB, which is required for efficient transcription of rRNA operons (Mata Martin et al., 2018).

A missing piece in the analogy of the eukaryotic nucleolus as a ribosome assembly line is the question where does ribosome assembly take place in a bacterium. 30S and 50S subunits of ribosomes can freely diffuse through the cells (Sanamrad et al., 2014) and thus their assembly could also take place in a non-constrained manner. By tracking single molecules of bacterial GTPases Era, needed for final steps of 30S subunit assembly, and of Obg, essential for large subunit maturation, we show that a fraction of slow-diffusing molecules exists for both proteins that diffuse mostly at polar sites in a constrained manner. This type of motion is abolished when RNA synthesis is blocked, showing that dependent on the supply of new RNA molecules, GTPases become engaged in ribosome maturation at places where translation takes place. Thus, our data suggest that as rRNA is transcribed at subpolar positions in the cell, close to the nucleoid borders, it diffuses towards the cell poles, where new ribosomal proteins are added and GTPases are bound to ensure proper maturation. This sets up an even stronger resemblance of subpolar regions to the nucleolus than previously thought.

Our SMT experiments show that Era and Obg are largely depleted from the central areas of cells and thus show NO in a similar manner to ribosomes. This important finding does not imply an absence of close spatial interaction with RNA polymerase (RNAP) molecules, which we have not studied in this work. Of note, using a resolution limit of 125 nm, it has been shown that 10 to 15% of ribosomes overlap with nucleoid borders, while about 4% RNAP molecules overlap with ribosome-dense areas close to the cell poles (Bakshi et al., 2012). It will be interesting to determine to what extent GTPases overlap with polar borders of nucleoids that contain the many rRNA operons. Single-molecule dynamics of Era and Obg revealed the existence of at least two diffusive states, one that corresponds to freely diffusing molecules, and one that comprises molecules showing slow motion, comparable to that of translating ribosomes. Assuming that the latter correspond to Era or Obg bound to ribosomal intermediates, about 40% of GTPases are engaged in this activity, while 60% are in search of new binding sites at the maturing complexes. Upon depletion of mRNA, the slow-mobile fraction sharply dropped and also strongly increased in its average diffusion constant. This resembles the changes observed in the dynamics of L1 (representing the ribosome), where mRNA depletion reduced the population of translating 70S/polysomes in favour of free subunits. Inhibition of transcription abolished NO for Era and strongly reduced the effect for Obg, similar for L1 and *Bs*S1. Inhibition of translation led to an increased NO for ribosomes. While ribosomes were stalled on their mRNA substrate, due to inhibition of peptidyl transferase activity, GTPases became more diffuse, likely because new supply of freshly synthesized rRNA and ribosomal proteins was lacking. Interestingly, we found that the stringent response overall retained NO for GTPases and for ribosomes. Although one would have expected that reduced supply of rRNA and inhibition of ribosome-associated factors by ppGpp (Corrigan et al., 2016) would have resulted in a pattern mimicking inhibition of transcription, organization of 70S ribosomes into polar clusters was retained. Also, there was only a small shift of 70S ribosomes towards free subunits, and likewise, Era populations did not shift, indicative of overall slower maturation of ribosomes but no loss of spatial organization, i.e., retained NO as in fast-growing, unperturbed cells. Only Obg showed a considerable loss of low-mobile/active molecules in favour of diffusing molecules, suggesting that for this protein, reduced amounts of substrate (new rRNA and r-proteins) leads to a change of its *in vivo* equilibrium. From these data, we propose that even during transient nutrient depletion, the spatial organization of ribosome maturation at the cell poles is largely retained.

Analyses of confined motion of GTPases, which represents molecules that are bound to much larger, less mobile structures, most likely GTPases bound to their substrate (i.e., maturing subunits) showed that NO is largely due to this fraction of molecules, rather than to freely diffusing GTPases. This seems coherent because free diffusion is not hindered by the nucleoids (Sanamrad et al., 2014; Rotter et al., 2021; Stracy et al., 2021), but accumulation of translating ribosomes leads to synthesis of ribosomal proteins at polar sites, and the association of slow-mobile GTPase molecules suggests that this process is directly coupled to the assembly of ribosome subunits. As a consequence, this further suggests that rRNA transcribed at nucleoid borders has to diffuse only a short distance towards the cell poles to become equipped with ribosomal proteins. Thus, our findings strongly support the idea of a nucleolus-like structure in bacterial cells containing nucleoids.

A hallmark of GTPases is their hydrolytic activity, shown to generate switch-like states that drive and regulate ribosome biogenesis. We investigated whether the induction of a mutant version of Era having a mutation in the GTP-binding motif that leads to loss of GTPase activity in Ras-like GTPases might disrupt the maturation process, hypothesizing that blocked GTPase activity would generate a dominant negative effect. To our surprise, we neither observed a noticeably effect on cell growth, nor on subcellular ribosome localization, when inducing a mutant allele from an inducible promoter. While we can nor prove that the mutation indeed affects GTPase activity of Era, it remains intriguing that high expression of this mutant protein does not affect the function of wild type Era expressed at much lower level.

Moreover, we show that the product of *ypfD*, which we propose to term “*Bs*S1” shows dynamics closely matching those of L1. *Bs*S1 was shown to be lost from ribosomes during purification and was therefore proposed not to be a ribosomal protein bound to the small subunit (Nanamiya et al., 2004). Our data suggest that while *Bs*S1 may frequently exchange binding at the 30S subunit, it can still be regarded as ribosome-associated component. NO of *Bs*S1 was visually less pronounced than that of L1, indicating higher mobility, however, the fraction of molecules that showed slow diffusion, like L1, was almost identical.

In summary, our single-molecule data reveal that slow-diffusive GTPases involved during ribosome biogenesis are excluded from nucleoids, similar to ribosomes in a translation-dependent manner. This observation implies that ribosome maturation takes place at sites of active translation and is not freely diffusive, unlike matured, single subunits of the ribosome. Altogether, this implies that translation and maturation are highly coordinated and occur close to sites where rRNA is mostly transcribed. This spatiotemporal organization is strikingly similar to the nucleolus, and thus could be seen as a case of convergent evolution of subcellular organization.

## Materials and methods

### Materials

Chemicals used in this work were purchased from AppliChem (Darmstadt, Germany), Carl Roth (Karlsruhe, Germany) or Sigma-Aldrich (Taufkirchen, Germany) unless stated differently.

### Strain construction

All fluorescently labelled strains were constructed by using a varied version of the integration vector pSG1164 (Lewis and Marston, 1999). The resulting strains contain a C-terminal mVenus-fusion of the desired proteins, amplified from the *Bacillus subtilis* genome, as the sole copy and under the original locus promoter. Cloning was done either by conventional cloning or by Gibson assembly (e.g., primers starting with GA; see Supplementary Table S5 for primers). The vector DNA (and PCR products) were digested with *Apa*I and *Kpn*I (all enzymes by New England Biolabs, United States).

In order to overproduce a point mutation as a second copy of the respective gene under control of a hyperspank promoter in the *amyE* site the pDR111 vector was used (Wagner et al., 2009). First era was amplified full length (primers: GA pDR111 era-fw, GA pDR111 era-rv) from the *Bacillus subtilis* genome and cloned into pDR111 *via Sal*I and *Sph*I restriction sites using the Gibson assembly method. The point mutation was introduced by amplification of two PCR products from the recent vector using both primer pairs (GA pDR111 eraN18A-fw/rv 1/2) before fusing both in a second PCR. The overlapping region contains the point mutation. Before Gibson assembly the vector DNA (*Sal*I and *Sph*I) and the PCR product (*Sal*I) were digested. All cloning steps were performed using DH5α *Escherichia coli* cells and correct vectors then were transformed into BG214 *Bacillus subtilis* cells. A list of all strains used for experiments in this work can be found in Supplementary Table S4.

For the visualization of the origin of replication, a LacI-*lacO* system was utilized. An array of *lacO* (Cm^r^) is intzroduced into the *Bacillus subtilis* chromosome at the *spo0J* site (359°, near the origin of replication) while the gene encoding for lactose repressor protein (*lacI*) is integrated into the genome *via* double crossover at the threonine locus (*thrC::lacI-cfp,* mls^r^) and is constitutively expressed (Webb et al., 1998; El Najjar et al., 2020).

### Cultivation

For *B. subtilis* cultivation cells were streaked from a cryo-stock on LB agar plates containing appropriate levels of antibiotics (5 μg/ml chloramphenicol and 100 μg/ml spectinomycin respectively) and afterwards grown as pre-cultures in LB medium with the same antibiotics. Incubation steps were carried out at 30°C and 200 rpm. For analysing the effect of a drug treatment, exponentially growing cells were incubated at 30°C with either rifampicin (40 μg/ml, 30 min), chloramphenicol (50 μg/ml, 30 min) or DL-serine hydroxamate (3.6 mg/ml ≈ 30 mM, 10 min) prior to tracking.

### Growth experiments

*Bacillus subtilis* pre-cultures were inoculated into fresh LB medium to yield an OD_600_ of 0.1. Each growth curve was measured as a biological triplicate from three different pre-cultures and as a technical triplicate for each culture. Cells were grown in a 96-well plate in volumes of 150 μl in a plate reader (Infinite**®** M Nano+, TECAN, Switzerland) with an orbital shaking radius of 2.5 mm at 30°C and 244 rpm. The OD_600_ was measured every 10 min as a triplicate readout.

### Survival assays

Pre-cultures of *Bacillus subtilis* cells were inoculated to fresh LB medium and incubated at 30°C and shaking at 200 rpm. When reaching an OD_600_ of 0.6 cells were diluted in serial dilutions of 1/10 of which 15 μl (dilutions 10^−2^ to 10^−7^) were plated on LB-Agar plates containing 1 mM of Isopropyl β-D-1-thiogalactopyranoside (IPTG). The plates were incubated at the stated temperatures of 22, 30 and 37°C.

### Epifluorescence microscopy

Pre-cultures were inoculated to fresh LB medium to an OD_600_ of 0.1 and let grown at 30°C until the cultures reach an OD_600_ of 0.6. For imaging, 5 μl of cell culture were placed on object slides and fixated with agar pads. For experiments involving DAPI staining exponentially growing cells were incubated with DAPI in a final concentration of 0.2 μg/ml for 10 min before the cells were placed on agar pads. Imaging was done on an Axio observer A1 (Zeiss) with an α Plan-Fluar 100x/1.45 Oil (Zeiss) and an Evolve EMCCD camera (Photometrics). Images were evaluated using ImageJ (Schneider et al., 2012).

### Single-molecule tracking microscopy

Pre-cultures of *B. subtilis* were inoculated to an OD_600_ of 0.1 in S7_50_ minimal medium with fructose as carbon source and 0.5% xylose for induction of downstream genes of the same operon, which are regulated by a *Pxyl* promoter. For the strains derived from the auxotrophic BG214 strain methionine and tryptophan, with a final concentration of 50 μg/ml each, were added to the minimal medium. Single-molecule microscopy experiments were performed using a Nikon Eclipse Ti-E (Nikon Instruments Inc) with a CFI Apochromat objective (TIRF 100x/1.49 Oil), an OBIS 514 nm LX 40 mW Laser (Coherent) and an ImagEM X2 EM-CCD camera (Hamamatsu). Movies were recorded with an integration time of 20 ms and only tracks with a length of at least 5 steps were included in the analyses. For processing of the acquired data ImageJ (Schneider et al., 2012), u-track 2.2.0 (Jaqaman et al., 2008) and SMTracker 2.0 (Rösch et al., 2018) were used.

Errors for diffusion coefficients and fraction sizes were calculated by using the cross-validation approach. Datasets were split into two parts, a training and a test group in a ratio of 70/30, and the training group was additionally split into 10 random subgroups for each of which a unique fit is done. Comparing the different subsets results in an average value ± the standard error of the mean + the 95% confidence interval which was obtained from the fit procedure (Oviedo-Bocanegra et al., 2021).

### Western blot

Pre-cultures of the desired strains were inoculated into fresh LB medium containing appropriate antibiotics and let grown to an OD_600_ of 0.6 at 30°C. Cells were then harvested (4°C, 4,000 x g) and resuspended with lysis buffer (50 mM EDTA, 100 mM NaCl) and lysed by the addition of lysozyme (2.5 mg/ml) at 37°C until the solution cleared up. Samples were diluted with SDS loading dye and incubated at 37°C for 1 h. The detection was done using an α-GFP antibody and goat-α-rabbit-IgG-HRP conjugate as second antibody. As protein size marker PageRuler™ Prestained Protein Ladder (Thermo Scientific) was applied to the gel.

### Ribosome isolation by sucrose density gradient centrifugations

Frozen pellets of harvested cells were then cryogenically ground to fine powder using a MM 400 mixer mill (Retsch) at a frequency of 30/s for 1 min. The pulverized material was then resuspended in Ribosome Buffer supplemented with DDM (25 mM HEPES pH 7.5, 150 mM KOAc, 30 mM Mg(OAc)_2_, 1 mM DTT and 0.025% DDM). The lysate was cleared at 4,000 x g at 4°C for 10 min and the supernatant was then pelleted through a 32% (w/v) sucrose cushion by centrifugation at 100,000 x g at 4°C for 16 h (70 Ti, Beckman Coulter). Crude ribosomes were resuspended in Ribosome Buffer and then 2 A_260 nm_ units were layered onto a 10–40% (w/v) linear sucrose gradient and centrifuged at 200,000 x *g* at 4°C for 4 h (SW40-Ti, Beckman Coulter). The gradient profile was then recorded using a Gradient Station (Biocomp) and the fractions corresponding to the 30S, 50S and 70S ribosomes were precipitated with 10% TCA, washed twice with 100% cold acetone, and resuspended in 2X SDS Sample Buffer (100 mM Tris–HCl pH 6.8, 4% w/v SDS, 0.2% bromophenol blue, 20% w/v glycerol, 200 mM β-mercaptoethanol) to proceed with Western blotting.

#### Importance

Many cells use membrane-surrounded compartments that facilitate physiological processes by spatially accumulating involved enzymes. We show that assembly of ribosomes in the model bacterium *B. subtilis* takes place at polar sites surrounding the centrally compacted chromosomes, where translation occurs, by visualizing the dynamics of GTPases involved in ribosome maturation. Thus, even in the absence of internal membranes, bacteria set up hot spots for particular processes, such as ribosome maturation.

## Data availability statement

The original contributions presented in the study are included in the article/Supplementary material, further inquiries can be directed to the corresponding author.

## Author contributions

JS performed all experiments (except for those shown in Figure 1), analyzed data, created constructs and strains, and wrote the manuscript. VZ performed experiments shown in Figure 1, and helped write the manuscript. GB helped write the manuscript, supervised experiments shown in Figure 1, and acquired funding. PG analyzed data, conceived of the study, and wrote the manuscript, and acquired funding. All authors contributed to the article and approved the submitted version.

## Funding

This work was supported by the Deutsche Forschungsgemeinschaft (TRR174) and by the state of Hessen (LOEWE Program for funding of SYNMIKRO).

## Conflict of interest

The authors declare that the research was conducted in the absence of any commercial or financial relationships that could be construed as a potential conflict of interest.

## Publisher’s note

All claims expressed in this article are solely those of the authors and do not necessarily represent those of their affiliated organizations, or those of the publisher, the editors and the reviewers. Any product that may be evaluated in this article, or claim that may be made by its manufacturer, is not guaranteed or endorsed by the publisher.
