# Supplementary material for: Single-molecule dynamics suggest that ribosomes assemble at sites of translation in Bacillus subtilis
Source: Front Microbiol. 2022 Nov 3;13:999176. doi: 10.3389/fmicb.2022.999176 (PMC9670183; doi:10.3389/fmicb.2022.999176)
Supplement: Supplementary file 1 [file Data_Sheet_1.pdf]

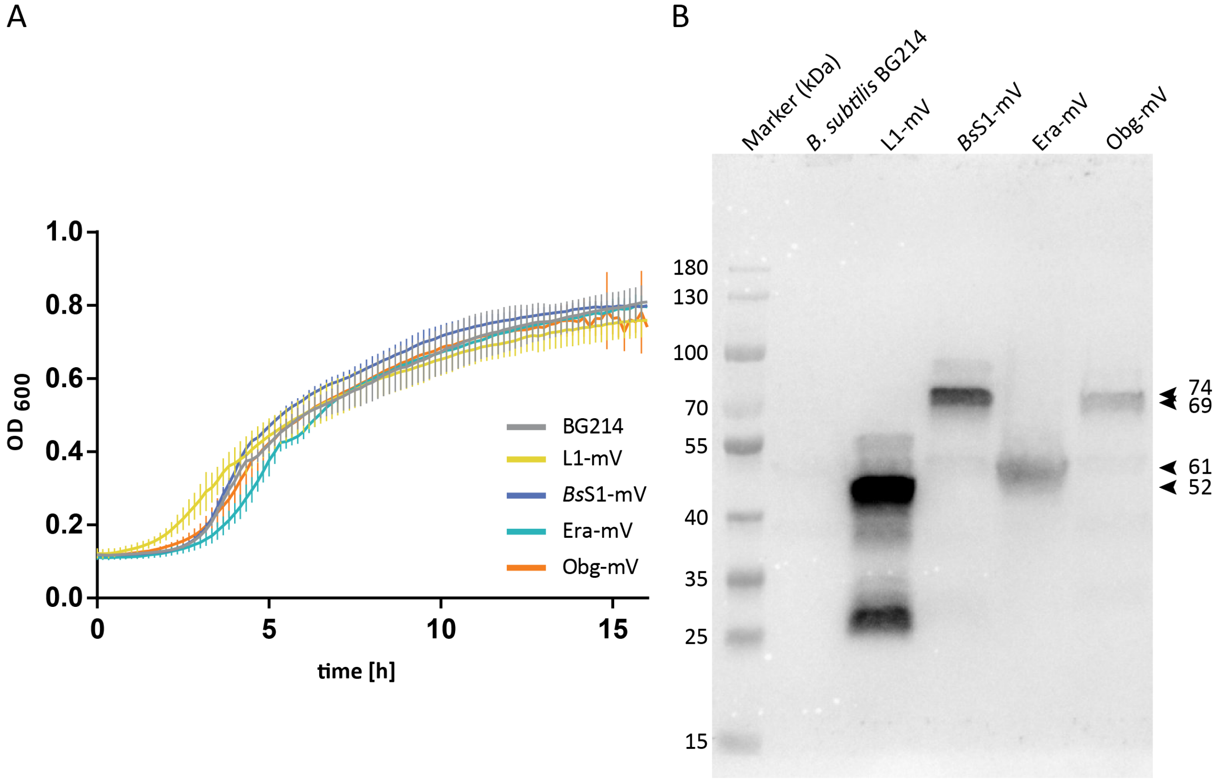

Figure S1. A) Growth curves of the strains used in this study. No changes in cell growth can be seen for the strains containing an mVenus fusion as the sole copy of the specific genes. The acquired data and their standard deviations are derived from a biological as well as a technical triplicate for each curve. B) Western blot of the strains when grown in fresh LB media to an OD_{600} of 0.6 inoculated from an overnight pre-culture. The detection was performed with an anti-GFP antibody. The expected sizes of the fusion proteins are L1-mV (51.7 kDa), *Bs*S1-mV (69.1 kDa), Era-mV (60.8 kDa) and Obg-mV (74.4 kDa). Note that there is a discrepancy between expected size and the detected bands, which most certainly derives from the fact that the samples in loading buffer were not boiled, because this treatment led to smearing of protein bands. For L1-mVenus a considerable amount of free mVenus protein was detected. Due to the fast diffusion constants of free fluorescent proteins, prior studies used integration times of 5 ms in order to track these molecules (Bakshi et al., 2012; Schibany et al., 2018), so free mVenus does not largely convolute acquired data.

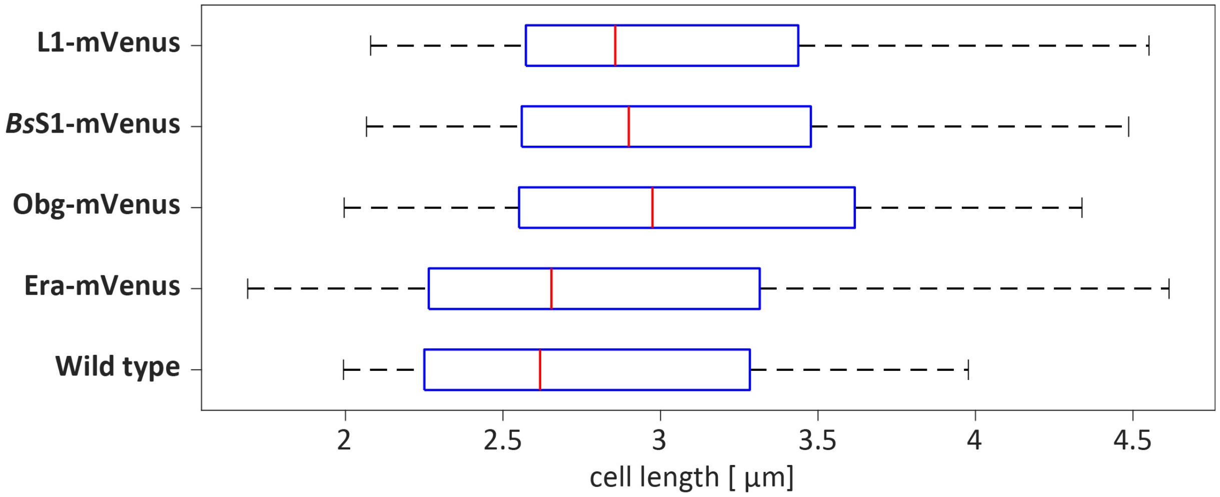

Fig. S2 Average cell lengths (red bar) determined by SMTracker 2.0. Boxes indicate 90% of all cell lengths, dotted lines outliers.

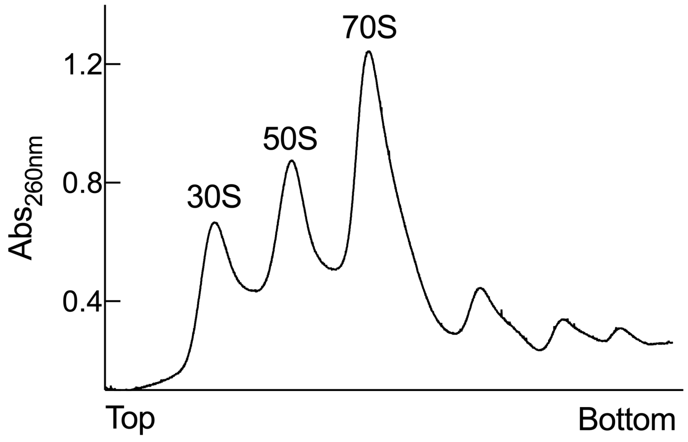

Fig. S3 Ribosome profiles of exponentially growing *B. subtilis* cells (wild type lacking any fusion construct). Peaks containing individual ribosomal proteins, translating 70S ribosomes or polysomes are indicated above the peaks.

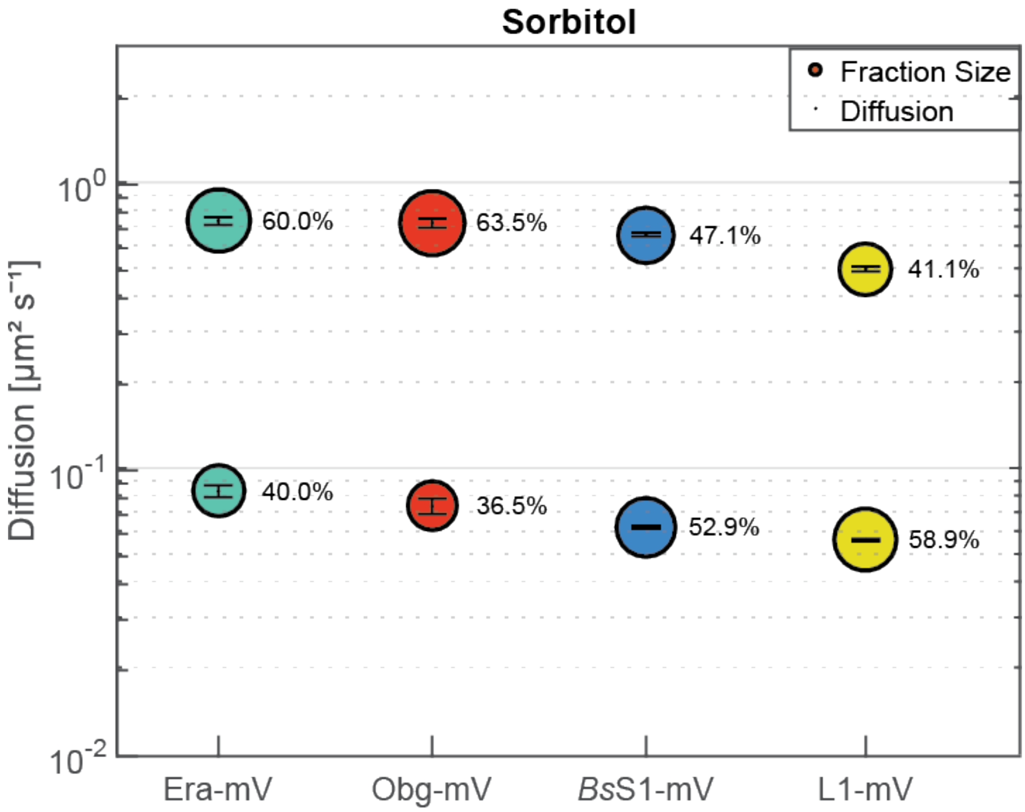

Figure S4. Bubble plot of the squared displacement analysis (SQD) for the sorbitol treatment experiment. This plot shows the diffusion constants and population sizes for a two-population fit. There are only minor changes (about 5%) in population sizes and diffusion speeds compared with normal growth conditions. A comparison with normal exponential growth can be found in table S3.

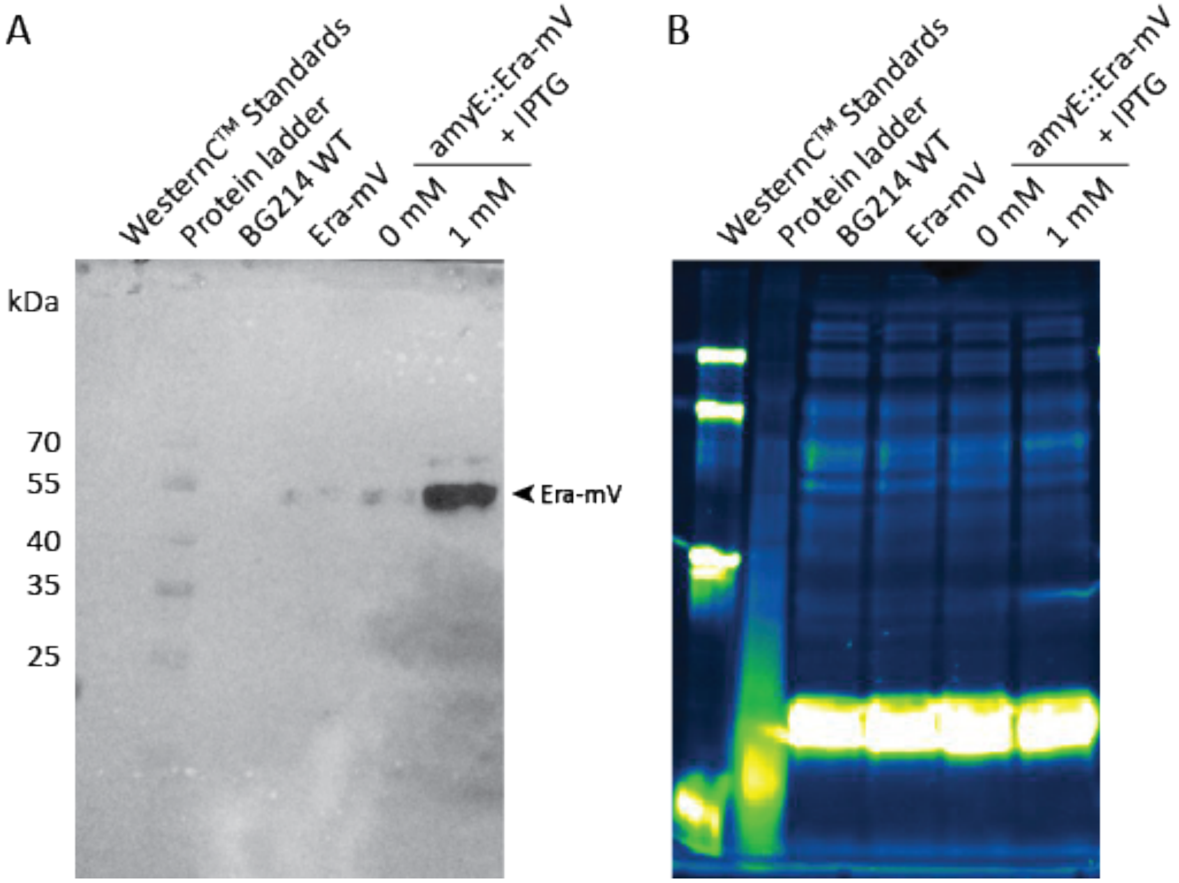

Figure S5 Western blot of the original locus Era-mV in comparison to the ectopically expressed gene under control of a hyperspank promoter induced (1 mM) and un-induced (0 mM). The expected gene size is approximately 62 kDa. As a negative control *B. subtilis* BG214 wild type was applied. A) For the sample preparation the lysates were incubated with SDS loading buffer at 37°C before running SDS-PAGE. Detection of the western blot was performed using an anti-GFP antibody. It becomes clear that even uninduced the hyperspank promoter is leaky and causes a protein level of Era-mV close to wild type level. B) The loading control was performed using a Mini-PROTEAN® TGX Stain-Free™ (Bio-Rad) and detection by UV light. This control reveals that the same amount of lysate was supplied for each pocket on the gel. Precision Plus Protein™ WesternC™ Standards was used as size standard for the loading control (excited by UV light). As size standard for the western blot the prestained protein ladder PageRuler™ was applied.

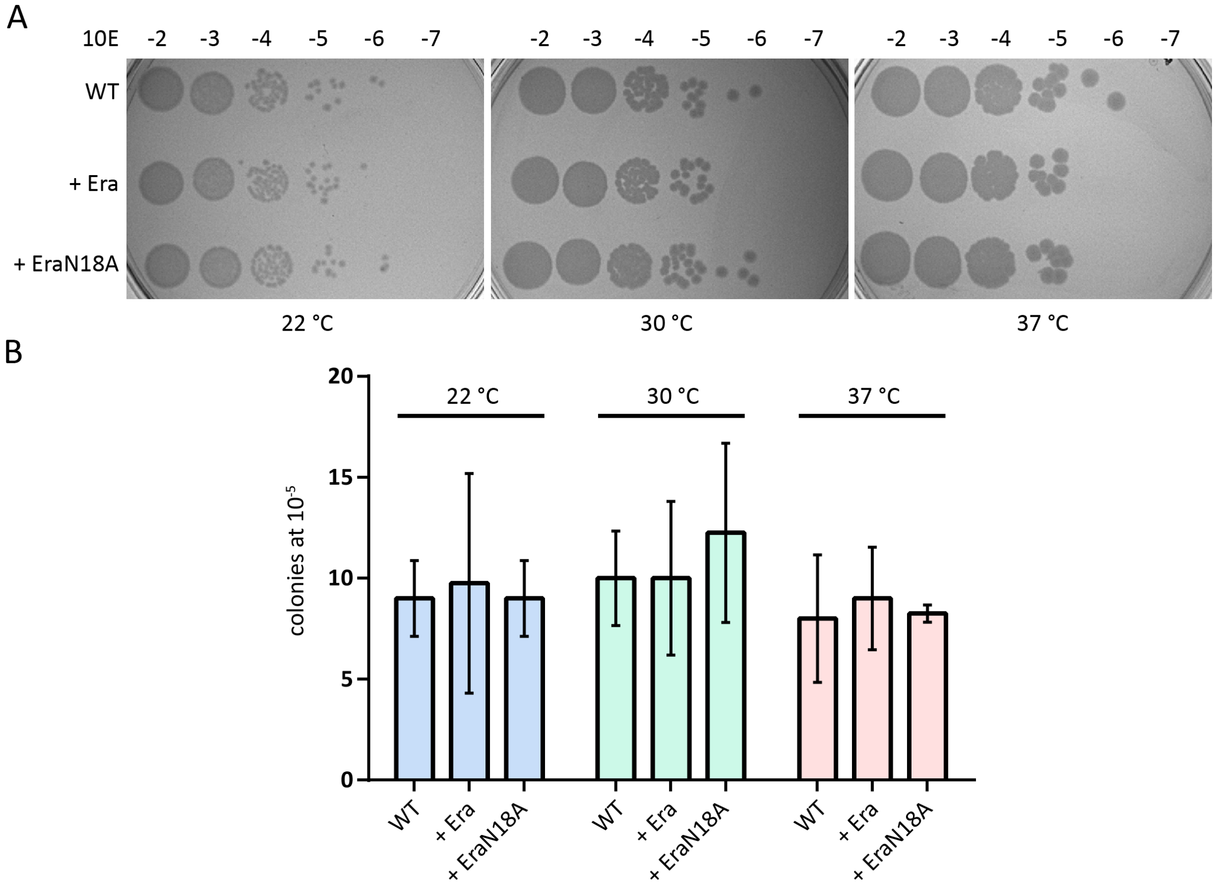

Figure S6 A) Spot assays of *B. subtilis* BG214 wild type cells and strains with ectopically expressed copies of wild type Era, or the EraN18A protein carrying a point mutation in the GTPase motif. Cells were grown as pre-culture to an OD_{600} of 0.6, diluted and plated afterwards. Incubation was performed at annotated temperatures. There was no significant difference in colony formation between the strains after expressing the ectopic genes. Experiments were performed as biological triplicate from different pre-cultures and as a technical duplicate each. B) Bar chart gives the number of colonies formed from 15 µl of a 10^{-5} dilution. Error bars represent standard deviation. Changes in relation to the wildtype strain are statistically not significant.

Table S1. Confinement radii used for confined motion maps. Note that maximum values of scales may change due to differences in the amount of overlayed track localization.

**Dwell radii [nm]**	**Era-mV**	**Obg-mV**	**YsxC-mV**	***Bs*S1-mV**	**L1-mV**
Exp. Growth	150	174	180	138	132
+ rif	210	192	177	216	177
+ cm	177	177	150	135	138
+ shx	192	204	195	159	132
**Scale max.**	**Era-mV**	**Obg-mV**	**YsxC-mV**	***Bs*S1-mV**	**L1-mV**
Exp. Growth	0.0142	0.0206	0.0460	0.0117	0.0131
+ rif	0.0179	0.0243	0.0359	0.0146	0.0295
+ cm	0.0236	0.0121	0.0436	0.0112	0.0150
+ shx	0.0242	0.0129	0.0099	0.0215	0.0134

Table S2. List of maximum values for heat map scaling from Fig. 6. A difference in scaling may result in a variation of coloration even though the total amount of tracks in separate maps is the same. The dwell radius cut was utilized for the creation of the confined maps shown in Fig. 6B.

**Heat maps**		**Era-V**	**Obg-mV**	***Bs*S1-mV**	**L1-mV**
Max. scale	small	0.0436	0.0632	0.0126	0.0118
	medium	0.0457	0.0150	0.0102	0.0160
	large	0.0311	0.0239	0.0103	0.0158
	combined	0.0292	0.0128	0.0097	0.0137
**Confined motion maps**		**Era-V**	**Obg-mV**	***Bs*S1-mV**	**L1-mV**
Max. scale		0.0541	0.0367	0.0090	0.0138
Dwell radius [nm]		220	177	156	126

Table S3. Diffusion constants and population sizes during growth in sorbitol-containing medium in comparison with values of cells growing at normal exponential rate.

**Exp. growth**	**Era-mV**	**Obg-mV**	***Bs*S1-mV**	**L1-mV**
pop_1 [%]	37.2 ± 1.0	42.6 ± 0.5	59.6 ± 0.6	58.6 ± 0.5
pop_2 [%]	62.8 ± 1.0	57.4 ± 0.5	40.4 ± 0.6	41.4 ± 0.5
D_1 [$\mu m^2 s^{-1}$]	0.081 ± 0.003	0.084 ± 0.001	0.052 ± 0.001	0.056 ± 0.001
D_2 [$\mu m^2 s^{-1}$]	0.57 ± 0.01	0.77 ± 0.01	0.59 ± 0.02	0.54 ± 0.01
**+ sorbitol**	**Era-mV**	**Obg-mV**	***Bs*S1-mV**	**L1-mV**
pop_1 [%]	40.0 ± 1.0	36.5 ± 2.0	52.9 ± 0.5	58.9 ± 0.5
pop_2 [%]	60.0 ± 1.0	63.5 ± 2.0	47.1 ± 0.5	41.1 ± 0.5
D_1 [$\mu m^2 s^{-1}$]	0.085 ± 0.004	0.075 ± 0.005	0.063 ± 0.001	0.057 ± 0.001
D_2 [$\mu m^2 s^{-1}$]	0.75 ± 0.02	0.74 ± 0.03	0.67 ± 0.01	0.51 ± 0.01

Table S4. Strains used in this work.

Strain / Organism	Genotype	Reference(s)
Bacillus subtilis BG214	*trpCE metA*5 *amyE*1 *ytsJ*1 *rsbV*37 *xre*1 *xkdA*1*att*^{SPß} *att*^{ICEBs1}	Juan C. Alonso (Madrid) lab strain collection
PG26 / *B. subtilis*	*lacO* cassette @ 359°, *thr::lacI cfp*, cmR, mlsR	Grauman lab strain collection
PG2724 / PY79	*era-mVenus* (original locus), cmR	Grauman lab strain collection
JS1	*era-mVenus* (original locus), cmR, parent BG214	This work
JS2	*obg-mVenus* (original locus), cmR, parent BG214	This work
JS3	*ypfD-mVenus* (original locus), cmR, parent BG214	This work
JS4	*rplA-mVenus* (original locus), cmR, parent BG214	This work
JS5	*era-mVenus* (original locus), *Phyperspank amyE*::*era*N18A, specR, cmR, parent BG214	This work
JS6	*rplA-mVenus*, (original locus), *Phyperspank amyE*::*era*N18A, specR, cmR, parent BG214	This work
JS7	*Phyperspank, amyE*::*era*N18A, specR, parent BG214	This work
JS8	*Phyperspank, amyE*::*era,* specR, parent BG214	This work
JS9	*Phyperspank, amyE::era-mVenus,* specR, parent BG214	This work
JS10	*ysxC-mVenus* (original locus), cmR, parent BG214	This work

Table S5. Primers used for cloning of the strains shown in table S4.

**Primers**	**5' – 3' sequence**
obg-fw	ATGGGTACCGAATTCCCATATGATGATTATC
obg-rv	GCCGGGCCCATCAATAAATTCAAATTC

GA ypfd-fw	GATTCCTAGGATGGGTACCGTTGATATTGGCGGCATCGAC
GA ypfd-rv	CCAGATAGGCCTTGGGCCCGTTTTAGTTTATTCAGCTTATCTCCGATC
GA rpla-fw	GATTCCTAGGATGGGTACCGCAAACGGAACTGGTAAAACTC
GA rpla-rv	CCAGATAGGCCTTGGGCCCGTTTTACGTTAAAAGTTGAAGAG
ysxC-fw	ACCGAATTCAAAGTCACAAAGTCAG
ysxC-rv	GCCGGGCCCCCGGTTTATCATTTTTTTG
GApDR111 era-fw	AGCTTAGTCGACAGGAGGACAAACATGACGAACGAAAGC
GApDR111 era-rv	TATCGCGCATGCTTAATATTCGTCCTCTTTAAAG
GApDR111 mVenus-rv	CACCGAATTAGCTTGCATGCTTACTTGTACAGCTCGTCCATG
GApDR111 eraN18A-fw1	TATTCACGAACGAAAATCGCCATTCGCCAGCTGCAGGAATTCGACCTC TAGCTTGAG
GApDR111 eraN18A-rv1	CAAAAATGTAGATTTTCCTACTGCTGGTCTTCCAATAATGGATAC
GApDR111 eraN18A-fw2	TATCCATTATTGGAAGACCAGCAGTAGGAAAATCTACATTTTTG
GApDR111 eraN18A-rv2	CACCGAATTAGCTTGCATGCTTAATATTCGTCCTCTTTAAAGC
